# A novel, complex RUNX2 gene mutation causes cleidocranial dysplasia

**DOI:** 10.1186/s12881-017-0375-x

**Published:** 2017-02-07

**Authors:** Wen’an Xu, Qiuyue Chen, Cuixian Liu, Jiajing Chen, Fu Xiong, Buling Wu

**Affiliations:** 1 Department of Stomatology, Nanfang Hospital, College of Stomatology, Southern Medical University, Guangzhou, Guangdong China; 2 Department of Stomatology, Zhongshan City People’s Hospital, Zhongshan, Guangdong China; 3 Department of Medical Genetics, School of Basic Medical Sciences, Southern Medical University, Guangzhou, Guangdong China

**Keywords:** Craniofacial anomalies, Oral systemic disease(s), RUNX2, Molecular genetics, Haploinsufficiency, Truncation protein

## Abstract

**Background:**

Haploinsufficiency of the runt-related transcription factor 2 (RUNX2) gene is known to cause cleidocranial dysplasia (CCD). Here, we investigated a complex, heterozygous RUNX2 gene mutation in a Chinese family with CCD and the pathogenesis associated with the variations.

**Methods:**

Genomic DNA extracted from peripheral venous blood was taken from the proband, her parents and 3 siblings, and 150 normal controls. Analysis of their respective RUNX2 gene sequences was performed by PCR amplification and Sanger sequencing. Pathogenesis associated with RUNX2 mutations was investigated by performing bioinformatics, real-time PCR, western blot analysis, and subcellular localization studies.

**Results:**

We identified 2 complex heterozygous mutations involving a c.398–399 insACAGCAGCAGCAGCA insertion and a c.411–412 insG frameshift mutation in exon 3 of the RUNX2 gene. The frameshift mutation changed the structure of the RUNX2 protein while did not affect its expression at the mRNA level. Transfection of HEK293T cells with a plasmid expressing the RUNX2 variant decreased the molecular weight of the variant RUNX2 protein, compared with that of the wild-type protein. Subcellular localization assays showed both nuclear and cytoplasmic localization for the mutant protein, while the wild-type protein localized to the nucleus.

**Conclusions:**

Our findings demonstrated that the novel c.398–399insACAGCAGCAGCAGCA mutation occurred alongside the c.411–412insG frameshift mutation, which resulted in RUNX2 truncation. RUNX2 haploinsufficiency was associated with CCD pathogenesis. These results extend the known mutational spectrum of the RUNX2 gene and suggest a functional role of the novel mutation in CCD pathogenesis.

## Background

Cleidocranial dysplasia (CCD, OMIM 119600) is an autosomal dominant human skeletal disorder resulting from haploinsufficiency of the Runt-related transcription factor 2 (RUNX2) gene, a master regulator for bone and cartilage development and maintenance [1–6]. CCD is characterized by a myriad of skeletal abnormalities and short stature. Skeletal abnormalities associated with CCD include hypoplastic or aplastic clavicles, patent sutures and fontanelles, dental abnormalities, and other skeletal abnormalities [2, 7, 8].

RUNX2 haploinsufficiency causes CCD and, although most CCD patients have a family history of CCD, approximately one-third of CCD patients were found to lack RUNX2 mutations [4, 9]. Here, we investigated a Chinese patient with CCD and identified 2 complex heterozygous RUNX2 mutations. To investigate the function and potential pathogenic mechanism of the RUNX2 mutant, we performed bioinformatics, real-time PCR, western blot analysis, and subcellular localization studies. Our results suggested that the novel mutations changed the molecular weight, structure, nuclear localization, and expression of the RUNX2 protein.

## Methods

### Patients

The proband (patient II-1), a 17-year-old girl, was referred to the Department of Stomatology at Nanfang Hospital for consultation regarding a dental abnormality. An experienced pediatric dentist performed clinical examinations for the proband and her family. Medical histories were obtained from the family members, including her parents and 3 siblings. One hundred and fifty normal controls from healthy individuals matched for gender and ethnic origin were recruited from Nanfang Hospital in Guangzhou, Guangdong. All subjects gave informed consent and the study was approved by the Ethics Committee of Southern Medical University.

### RUNX2-gene mutation screening

To identify disease-associated mutations, we extracted genomic DNA from the peripheral blood of the proband and her family members by a standard phenol/chloroform extraction method. PCR reactions were performed using primers designed with Primer3 plus software, the sequences of which are shown in Table 1. The PCR products were visualized by 1.5% agarose gel electrophoresis and subsequently analyzed by Sanger sequencing.

**Table 1:** Primers used in RUNX2polymerase chain reaction (PCR)

Positions	Forward nucleotide sequence (5′-3′)	Reverse nucleotide sequence (5′-3′)
Exon 1	AGAGAGAGAAAGAGCAAGGGG	GCATAGACTGTGGTTAGAGAGC
Exon 2	TTTCTTTGCTTTTCACATGTTACC	TGCTATTTGGAAAAGCTAGCAG
Exon 3	CGCTAACTTGTGGCTGTTGT	CGTGGGCAGGAAGACACC
Exon 4	CATTCCTGTCGGCCATTACTG	CATCAAAGGAGCCTAATGTGCT
Exon 5	AAGTGGTCATCGGAGGGTTT	TGCAGATAGCAAAGTCCACAA
Exon 6	GGCCACCAGATACCGCTTAT	CCAGCGTCTATGCAAGTGAA
Exon 7	GCCTGAAAGGATGGGGTTAT	CTGTGCAGGGATGGATTTTT
Exon 8	CTTATGGGCCTGCAGACTCT	AGTAACAACCAGACAGCCCA
Exon 9	CTGTGGCTTGCTGTTCCTTT	TGATACGTGTGGGATGTGGC

To identify RUNX2 (NCBI Reference Sequence: NM_001015051.3) gene mutations, PCR products corresponding to exon 3 of RUNX2 were cloned into the PMD-18 T vector (TaKaRa Biotechnology, Dalian, Co., Ltd) and introduced into DH5α bacteria (TaKaRa Biotechnology). Transformants were then isolated, and the RUNX2 gene sequences were studied by PCR and DNA sequencing.

### RNA analysis

Total RNA was extracted from the peripheral blood of the proband and her parents with TRIzol (Invitrogen, Carlsbad, CA, USA) and purified by chloroform extraction and isopropanol precipitation. Total RNA samples were quantified by measuring the absorbance at 260 and 280 nm. Reverse transcriptase-polymerase chain reactions (RT-PCR) were performed using the PrimeScript RT-PCR Kit (TaKaRa Biotechnology). Quantitative RT-PCR (qRT-PCR) was performed to compare peripheral blood RUNX2 mRNA expression levels between the patient and her parents. qRT-PCR was performed using Platinum SYBR Green (Bio-Rad Laboratories, California, USA) and an MxPro Real-Time PCR System (Stratagene MX3005P), using 40 cycles of 95 °C for 20 s, 63 °C for 20 s, and 72 °C for 20 s. The sequences of the primers used for the qRT-PCR experiments are shown in Table 2. Each sample was analyzed in triplicate, and β-actin mRNA expression was measured as a reference. Student’s 2-tailed *t*-test was used for statistical analysis.

**Table 2:** Primers used for qRT-PCR

Positions	Forward nucleotide sequence (5′-3′)	Reverse nucleotide sequence (5′-3′)
q-RUNX2	TCCTCCCCAAGTAGCTACCT	GAGGCGGTCAGAGAACAAAC

### Bioinformatics

Three-dimensional structures of the wild-type and mutant RUNX2 protein were predicted using the I-TASSER server [10, 11].

### Construction of recombinant plasmids

PCR fragments encoding the mutant or wild-type RUNX2 gene were amplified using primers designed by Oligo 7 (sequences shown in Table 3) to have an annealing temperature of 63 °C. The PCR products were extracted from a 1.5% agarose gel using the SanPrep Column DNA Gel Extraction Kit (Sangon Biotech, Shanghai). The purified PCR product for the mutant gene was then cloned into the PMD-20 T vector (TaKaRa Biotechnology) to generate the PMD-20 T-RUNX2 mutant construct. After sequence confirmation, the inserted mutant RUNX2 gene was then subcloned into the pEGFP-C1 vector via the SalI and XamI restriction sites, generating a recombinant plasmid (pEGFP-C1-RUNX2) encoding the mutant RUNX2 gene. A recombinant plasmid encoding the wild-type RUNX2 gene was constructed using the same method.

**Table 3:** Primers designed by oligo 7

Name	Forward nucleotide sequence (5′-3′)	Reverse nucleotide sequence (5′-3′)
RUNX2	ATGGCATCAAACAGCCTCTTC	TCAATATGGTCGCCAAACAGA

### Western blot analysis

To study protein expression, recombinant plasmids encoding wild-type or mutant RUNX2 templates, as well as the parental vector (pEGFP-C1), were transfected separately into human embryonic kidney (HEK) 293 T cells. After 24 h in culture, total proteins were extracted with cell lysis buffer for western blotting (Beyotime, Shanghai) containing of Protease Inhibitor Cocktail (Sigma, St. Louis, MO). The proteins were resolved by sodium dodecyl sulfate-polyacrylamide gel electrophoresis. The separated proteins were transferred to polyvinylidene difluoride membranes (Millipore). After the membranes were blocked in TBST containing 5% non-fat milk, they were incubated overnight with a primary antibody against enhanced green fluorescence protein (Santa Cruz Biotechnology, USA). Next, the membranes were incubated for 2 h with an appropriate secondary antibody (Santa Cruz Biotechnology) at room temperature. Relative RUNX2 protein expression levels were calculated after normalization to glyceraldehyde 3-phosphate dehydrogenase (Santa Cruz Biotechnology) expression. Protein expression was detected using the Immobilon Western Chemiluminescent HRP Substrate (Thermo Fisher, USA).

### Cell localization studies

Recombinant plasmids encoding wild-type or mutant RUNX2 genes were transfected into COS7 cells to study the subcellular localization of the RUNX2 protein and the impact of the mutation. At 48 h post-transfection, cells were rinsed 3 times with PBS, and nuclei were stained with 0.1 μg/ml 4′,6-diamidino-2-phenylindole (Sigma) for 10 min at room temperature. Subsequently, the cells were viewed under an Eclipse Ti-U fluorescence microscope (Nikon; Tokyo, Japan).

## Results

### Clinical features of the proband patient

The patient underwent a detailed clinical evaluation by an experienced dentist. Physical examination revealed short stature (height: 143 cm), dental abnormalities, a high-arched palate, open fontanelles, and ptotic and hypermobile shoulders. The remaining clinical features tested for were normal. The family history and physical examinations revealed no other family members with bony abnormalities.

**Fig. 1:**
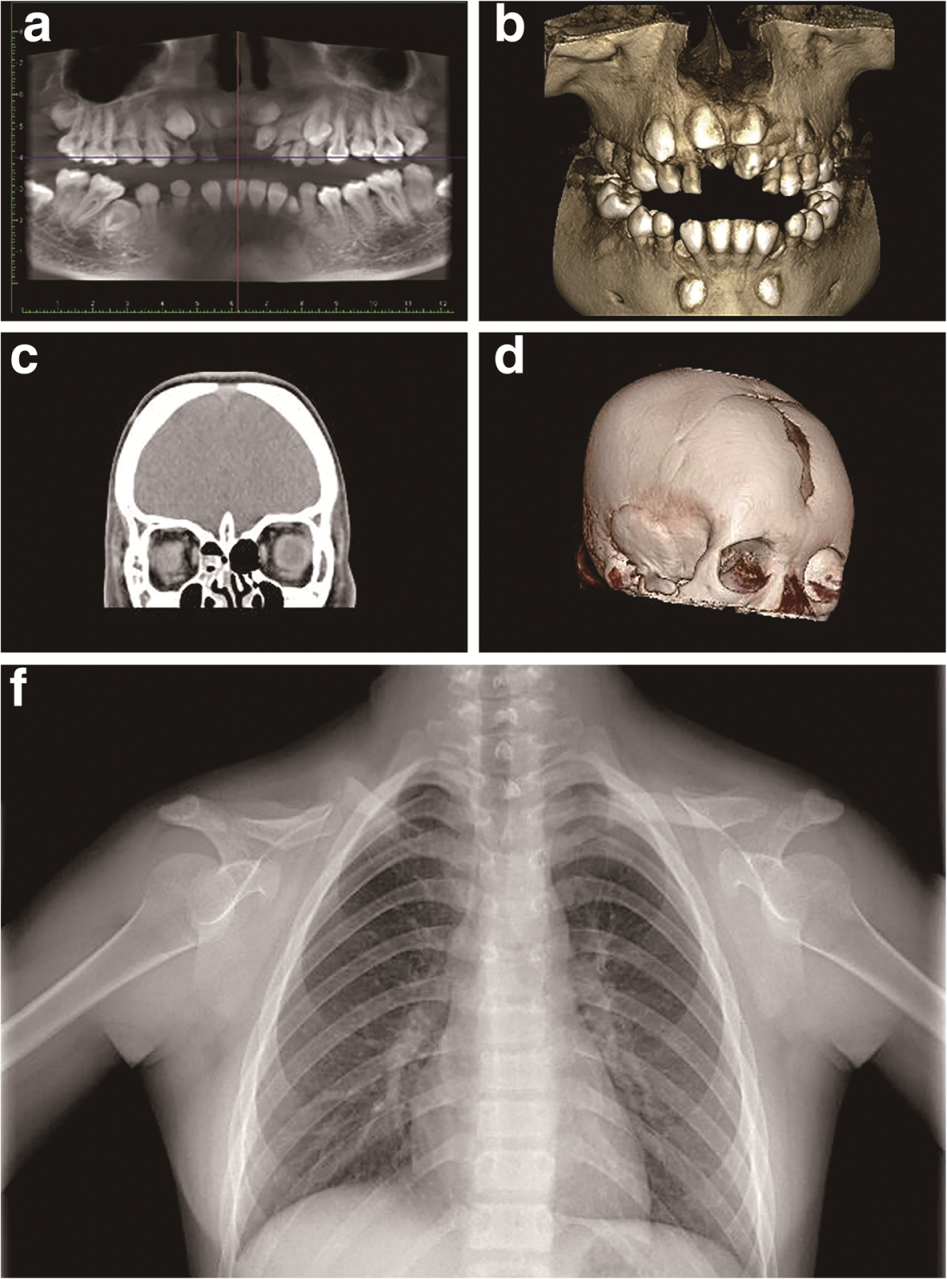
Radiological findings for the patient. **a**, **b** Cone-beam computed tomography results showing detailed dental abnormalities, including impacted supernumerary teeth, the retention of primary teeth, eruption failure of the permanent teeth, and impaired root development. **c**, **d** A skull CT scan showed the presence of open fontanelles. **e** Radiographs revealed hypoplastic or aplastic distal ends of clavicles and structural abnormalities occurring in the right shoulder peak joint

Detailed dental abnormalities, including impacted supernumerary teeth, retention of primary teeth, eruption failure of permanent teeth, and abnormal root development were very instructive for devising a treatment plan (Fig. 1a, b). Delayed closure of the cranial sutures and detailed dental abnormalities were identified by computed tomography (Fig. 1c, d). Chest radiographs showed hypoplastic or aplastic clavicles and structural abnormalities of the right shoulder-peak joint (Fig. 1e). The overall characteristics of the clinical and radiographic results supported a clinical diagnosis of CCD.

### Sequence analysis of RUNX2 gene

**Fig. 2:**
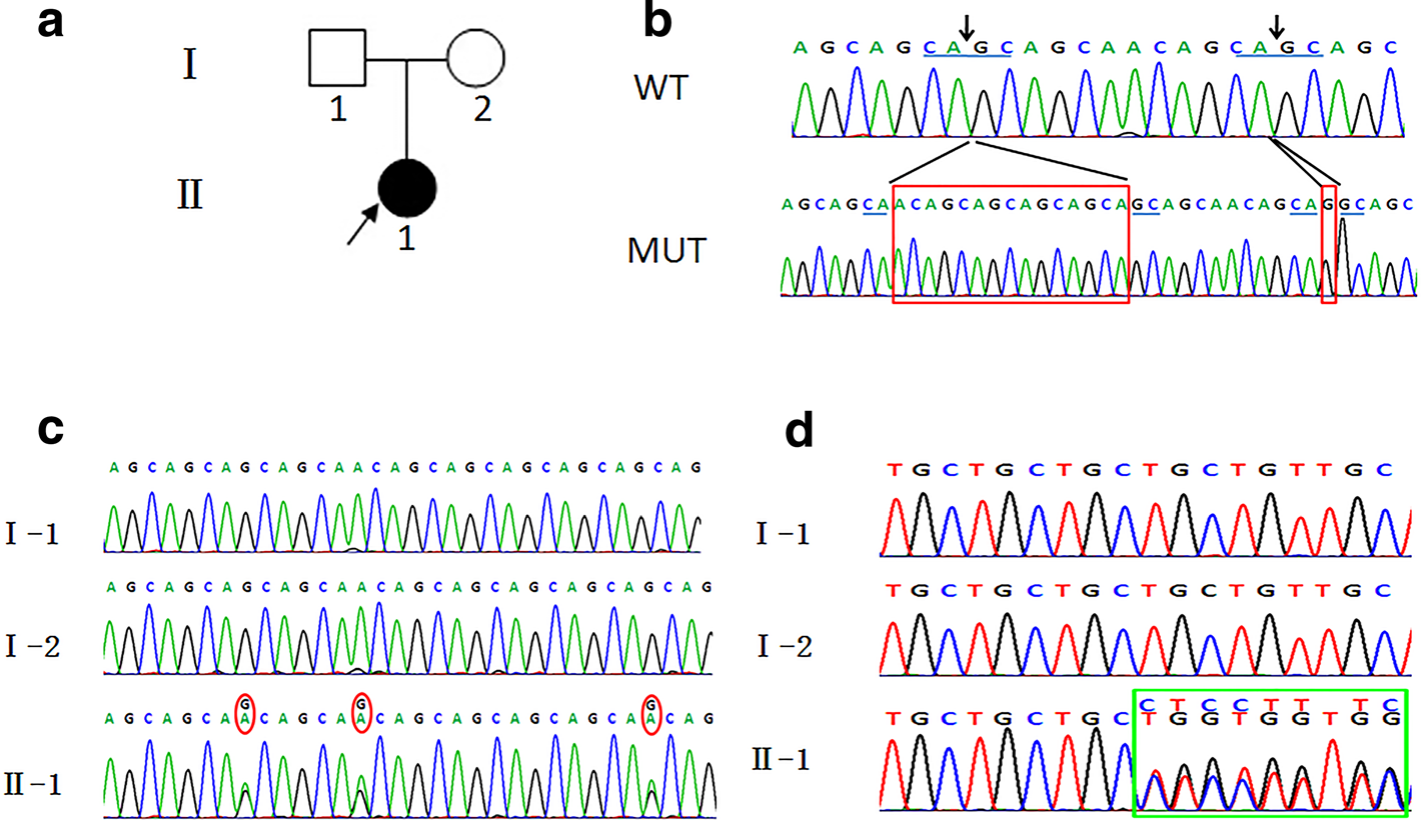
Mutation analysis. **a** Family pedigree. The arrow indicates the proband in the family. **b** Mutation screening. Sequences of the wild-type (WT) and mutant (MUT) RUNX2 gene sequences occurring in exon 3. Nucleotides highlighted in the red boxes show the insertion mutations. **c** Forward DNA sequencing results. Panels I-1 and I-2 show the sequences of wild-type RUNX2 alleles, and panel II-1 shows double peaks within this region. **d** Reverse DNA sequencing results. Panels I-1 and I-2 show sequences of WT RUNX2 alleles, and panel II-1 shows a double-peak phenomenon

All 9 coding exons of the RUNX2 gene were amplified by PCR. Direct screening revealed complex mutations in exon 3 of the patient’s gene, which were not identified in the parents’ DNA (Fig. 2b). Thus, these mutations arose de novo, as neither parent carried these mutations, and non-paternity was excluded by paternity testing.

BLAST analysis revealed novel mutations (c.398–399 insACAGCAGCAGC AGCA and c.411–412insG) in cis in the runt domain of the patient’s RUNX2 gene (Fig. 2c, d). The insertions created a frameshift mutation that led to the translated nornal proteins changing into truncated proteins containing 165 amino proteins. Both mutations were absent in DNA samples from 150 unrelated, normal control subjects that were matched for Chinese ethnicity to rule out the possibility of these mutations occurring naturally as single-nucleotide polymorphisms.

### Functional analysis

**Fig. 3:**
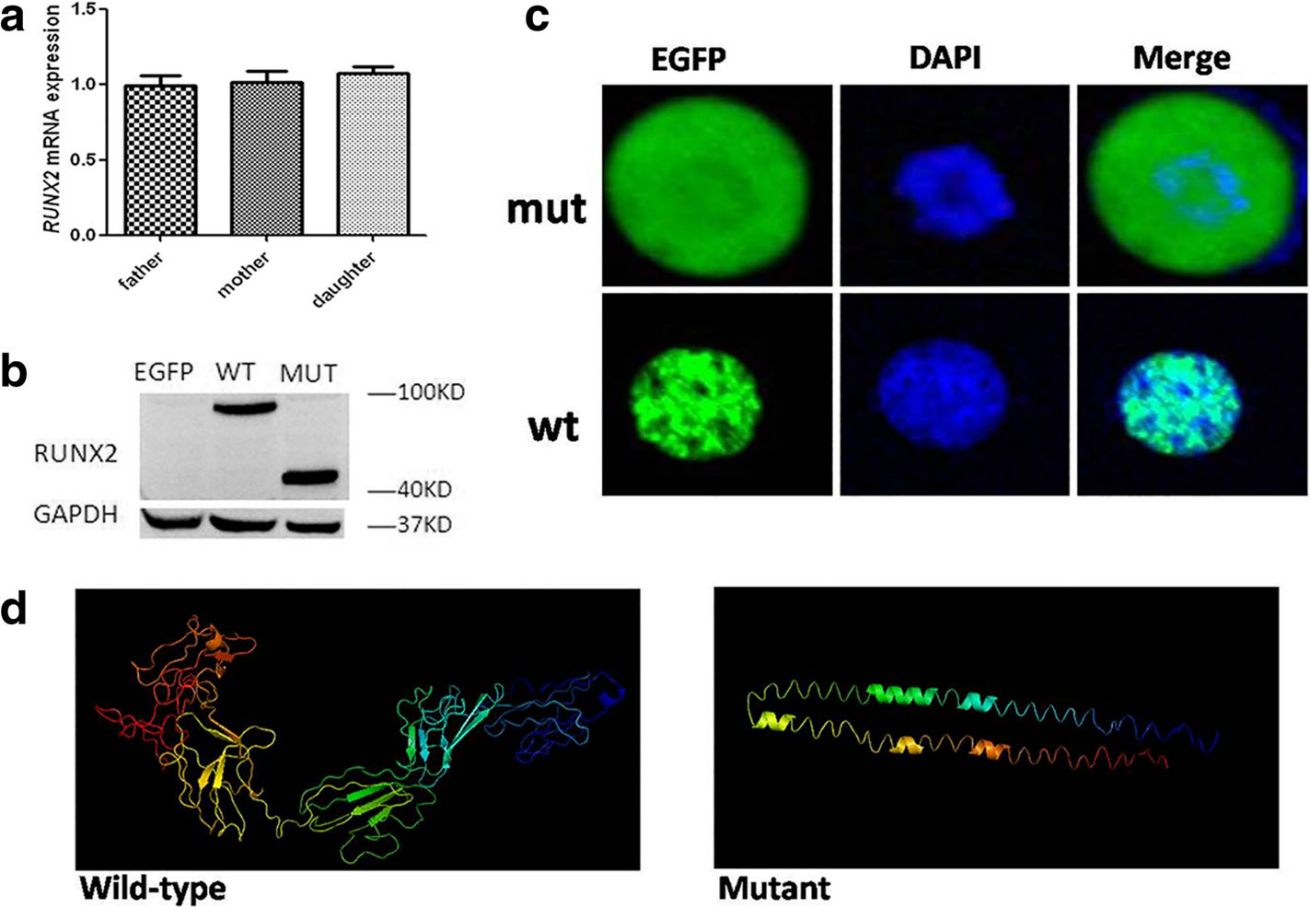
Functional analysis of wild-type and mutant RUNX2. **a** Quantification of RUNX2 mRNA expression levels revealed no significant difference (*P* = 0.6218) between the patient and her parents. **b** Western blot analysis of RUNX2 protein expression showed that transfection of the wild-type RUNX2 construct resulted in full-length RUNX2 protein production, whereas overexpression of mutant RUNX2 generated a truncated protein. GAPDH, 37 kDa. **c** COS7 cells were transfected with recombinant plasmids encoding the wild-type or mutant RUNX2 genes. Confocal micrographs showed the intracellular distributions of the wild-type and mutant RUNX2 proteins. **d** Molecular modeling performed using the I-TASSER server revealed that the mutant runt domain plays a critical role in the normal 3-dimensional structure of RUNX2, as indicated

Our qRT-PCR results showed that the patient’s RUNX2 messenger RNA expression level was not significantly different from those of her parents (Fig. 3a). However, western blot analysis revealed that transfection with a wild-type RUNX2 construct resulted in the production of a 56-kDa protein, whereas transfection with the mutant RUNX2 construct produced an 18-kDa, truncated protein (Fig. 3b). These molecular masses matched the expected sizes of the encoded RUNX2 variants. Three-dimensional structures predicted with the I-TASSER server showed the anticipated structural changes resulting from the RUNX2, exon 3 mutations (Fig. 3d).

The exon 3 mutations affected subcellular location of the RUNX2 variant. The mutant protein expressed from the pEGFP-C1-RUNX2 plasmid was distributed throughout both the cytoplasm and nucleus of transfected COS7 cells, whereas the wild-type protein was localized only in the nucleus (Fig. 3c).

## Discussion

Mutations in the RUNX2 gene, that maps to the short arm of chromosome 6, cause CCD [12]. The RUNX2 gene encodes a transcription factor that is a member of the core-binding factor family. In this report, we present data from a patient with a novel in-frame insertion mutation in the RUNX2 gene and CCD symptoms, such as disproportionate short stature and the classic triad of multiple supernumerary teeth, open sagittal sutures and fontanelles, and hypoplastic or aplastic clavicles [13, 14]. Apart from these typical clinical features, CCD patients can show other symptoms, such as mental deficiencies, hearing disorders, median pseudo-cleft palates, delayed ossification of the pelvis, and other skeletal abnormalities [15–17]. CCD is an autosomal dominant genetic disease; thus, patient’s parents generally harbor the same genetic mutation, although patients with unaffected parents have been reported in several studies [4, 9, 13]. In this study, insertional mutations were found in exon 3 of the patient’s RUNX2 gene, while her parents lacked these mutations. Paternity testing excluded the non-paternity between the proband and her parents, thereby indicating that the genetic abnormalities arose as de novo events. We propose that the de novo mutations should have arisen during the spermatogenic process or an early stage in embryonic development.

Although CCD-related bone anomalies develop through an unclear mechanism(s), it is known that CCD results from RUNX2-dependent signaling pathways. The RUNX2 protein regulates extracellular matrix properties and mineralization through transforming growth factor-β-responsive, COL10A1, VEGF, and MMP13 pathways, providing supporting evidence that RUNX2 affects endochondral ossification, intramembranous ossification, and chondrocyte maturation [16, 18, 19]. The RUNX2 protein forms a complex with core-binding factor β (CBFβ) and binds to a conserved nucleotide sequence (R/TACCRCA) to drive expression of several osteogenic proteins, such as collagen a1, osteopontin, bone sialoprotein, and osteocalcin [20, 21]. Collectively, these findings indicate that RUNX2 plays an important role in osteoblast differentiation. Periodontal ligament stem cells modulate root resorption in human primary teeth via the RUNX2-regulating, receptor activator of nuclear factor kappa-B ligand pathway, causing the retention of primary teeth [22]. The RUNX2 protein can be thought of as having 4 domains, consisting of the N terminus, the runt domain, the PST domain, and the C terminus. The runt domain and nuclear localization signal (NLS) at the C-terminal domain border is important for the transcriptional activity, subcellular distribution, and aggregation of the RUNX2 protein [23–25]. Furthermore, many findings have revealed that the runt domain is responsible for DNA binding and heterodimerization with the CBFβ protein [3, 4] The CBFβ protein is an unrelated binding partner that enhances the DNA-binding affinity of the RUNX2 protein [26].

In the present study, the patient showed normal RUNX2 mRNA expression compared with her parents, which indicated that the mutation did not affect mRNA transcription. However, the insertional mutation occurring in the patient led to premature translation termination, producing a truncated protein containing 165 amino proteins. Structural modeling suggested that the normal molecular structure of the truncated protein was altered, thereby abolishing the function of the runt domain, which could also redirect nuclear RUNX2 from the nucleus to the cytoplasm to prevent binding to CBFβ [24, 25]. In addition, the truncated protein lost the NLS, which is normally present at the C-terminal border. This alteration could impair the transcriptional activation function of RUNX2, resulting in decreased ossification and skeletal deformities [1, 23].

## Conclusions

Haploinsufficiency of the RUNX2 gene is known to be the reason of CCD. Here, we present a molecular cytogenetic characterization of novel RUNX2 insertional mutations occurring in a 17-year-old female patient with CCD and discuss potential genotype–phenotype correlations in this case. Data presented here expand the known RUNX2 mutation spectrum and potentially shed insight into the development of CCD.

## Abbreviations

CBFβ: Core-binding factor β
CCD: Cleidocranial dysplasia
HEK: Human embryonic kidney
NLS: Nuclear localization signal
qRT-PCR: Quantitative RT-PCR
RT-PCR: Reverse transcriptase-polymerase chain reactions
RUNX2: Runt-related transcription factor 2
